# Path Analysis of the Relationships between the Eruption Time of the First Primary Teeth and Various Factors in Twins

**DOI:** 10.3390/children10040683

**Published:** 2023-04-04

**Authors:** Sinem Birant, Mert Veznikli, Yelda Kasimoglu, Mine Koruyucu, Atıf Ahmet Evren, Figen Seymen

**Affiliations:** 1 Department of Pedodontics, Faculty of Dentistry, Istanbul University-Cerrahpasa, Istanbul 34098, Turkey; 2 Department of Statistics, Faculty of Science and Arts, Yildiz Technical University, Istanbul 34220, Turkey; 3 Department of Pedodontics, Faculty of Dentistry, Istanbul University, Istanbul 34452, Turkey; 4 Department of Pedodontics, Faculty of Dentistry, Altinbas University, Istanbul 34218, Turkey

**Keywords:** birth weight, breast feeding, child, dizygotic, genetic factors, moderating effect, monozygotic, multi-group analyses, partial least squares, path analysis, structural equation model, tooth eruption age, twins

## Abstract

The timing of primary tooth eruption is critical for children’s health planning and the diagnosis of specific growth disorders. The purpose of this study is to assess the relationship between twin pairs’ birth weight, gestational age, and gender, which are indicators of prenatal factors; breast-feeding duration, which is an indicator of postnatal factors; type of delivery, which is an indicator of maternal as well as genetic factors; and age of the primary tooth. Twin children aged from 3 to 15 years who applied to the clinic for the first dental examination constituted the sample group. In this twin study, 59 monozygotic (MZ) twin pairs and 143 dizygotic (DZ) twin pairs were included. Genetic (MZ vs. DZ), maternal (type of delivery, gestational age), perinatal (birth weight, gender), and postnatal (duration of breastfeeding) information was obtained, and effects on the children’s Eruption Timing of the First Primary Tooth (ETFPT) were examined. Statistical analysis was performed using the consistent partial least squares structural equation model (robust PLSc) technique. As birth weight increased, the age at first eruption became younger, but this change was different between MZ and DZ twins (*p* < 0.05). While the age at first tooth eruption was older in identical twins who were breastfed for the first 6 months, this increase was not observed in DZ twins. The mean of ETFPT was calculated as 7.31 months in MZ twins and 6.75 months in DZ twins. The effect of breastfeeding and birth weight on ETFPT may differ according to zygosity in twins. MZ twins may tend to take longer to experience the eruption of their first primary teeth.

## 1. Introduction

Tooth eruption is the progressive migration of a tooth from the alveolar bone’s developing zone to the location where it will function in the mouth and the occlusal plane [1]. Tooth emergence into the oral environment is a brief moment in the ongoing dynamic process of tooth eruption, when the tooth pierces through the oral epithelium [2]. The eruption time of the primary teeth is of great importance for the growth and development of the child [1,3]. The eruption of primary teeth is critical for the appropriate alignment and occlusion of permanent teeth, child health planning, and the identification of various growth abnormalities [4]. The calcification of the primary teeth begins on average in the fourth month of the prenatal period, and all primary teeth have begun to calcify by the end of the sixth month of the prenatal period. The eruption of all primary teeth is usually experienced between the ages of 24 and 36 months. The formation, calcification stage, and eruption process of primary teeth depend on individual factors but are also affected by many other variables [4]. According to the literature, the eruption timing of primary teeth is genetically determined. However, perinatal maternal circumstances, early childhood metabolic, systemic, and nutritional factors, such as the type of feeding, socioeconomic variables, and ethnicity are also all linked to primary tooth eruptive timing [2,3,5,6,7,8,9]. The effect of gender differences on the eruption times of primary teeth has not been clearly determined. There are various opinions about the influence of the infant’s gender [3,4,10,11] including the conventional view that girls generally have earlier eruptions of primary teeth than boys [3]. Birth weight is an indicator of perinatal factors, and it has been reported in the literature that birth weight is associated with age at the eruption of primary teeth [2,4,12]. There is a positive correlation between late eruption and low birth weight due to the general developmental delay in these children [9]. This supports the assumption that malnourished children in the prenatal period, when teeth are just beginning to form, suffer from nutritional problems that can negatively impact the development of their primary teeth [1,9]. In terms of chronological age, the eruption time of primary teeth was delayed in preterm children, and this was found to be related to non-breastfeeding and very low birth weight [13]. Breastfeeding is one of the postnatal components that affect the eruption time of primary teeth. Breast milk is the ideal nutrition source for infants; it contains antibodies that increase the baby’s immunity, and it plays a role in the natural development of a healthy oral environment [3,14]. Early breastfeeding activity influences craniofacial complex growth and development, as well as the physiological development processes of both musculoskeletal and skeletal components. In addition, the act of breastfeeding promotes the proper growth and development of the mouth and jaws, as well as the secretion of appropriate hormones for the digestive system [3,14,15]. Breastfeeding provides the main nutrients needed for children in the first six months of life and can thus have a significant impact on growth and development throughout this time. It was suggested that exclusive breastfeeding may have an effect on tooth emergence [16]. However, there is no conclusive evidence to date on the possible effects of breastfeeding on the timing of dental eruptions in children.

Preterm birth, which is one of the perinatal factors, can affect the child’s oral growth and development [17]. Many studies have shown that preterm birth delays the Eruption Timing of the Primary Tooth (ETFPT) [17,18,19]. Primary teeth eruption time in children born prematurely was found to be delayed and associated with very low birth weight in a recent systematic review [12].

Delivery is one of the maternal factors affecting tooth development. The neonatal line is accepted as an indicator of birth and is formed as a result of a disruption in mineralization reflecting physiological trauma at birth [20]. It has been thought that the delivery type may also have effects on tooth development, and many studies have been conducted on this subject. The effects of delivery type on the eruption timing of primary teeth have not been fully explained.

The present study was undertaken to determine whether the relationships among prenatal, postnatal, and maternal factors and age at the time of eruption of primary teeth differ between monozygotic (MZ) and dizygotic (DZ) twin pairs. This study explores the effects of gestational age, gender, birth weight, breastfeeding duration, mode of delivery, and zygosity on the eruption time of the first primary tooth (ETFPT).

## 2. Materials and Methods

### 2.1. Study Design

In this twin study, it was intended to assess the relationships among maternal, perinatal, and postnatal components as well as genetic factors and ETFPT among twin siblings.

#### 2.1.1. Samples Collection

The study sample comprised only MZ and DZ pairs of twins between the ages of 3 and 15 years, who applied to the pedodontic clinic of Istanbul University, Faculty of Dentistry, for an oral examination during 2014–2017 without any systemic or genetic diseases, mental problems, or previous orthodontic treatments. Triplets and quadruplets were not included in this study.

#### 2.1.2. Ethical Approval

The Istanbul University, Istanbul Faculty of Medicine Clinical Research Ethics Committee granted ethical approval with Approval Code 592 in accordance with the Helsinki Declaration.

### 2.2. Zygosity Determination

The zygosity status of all twin pairs was initially recorded according to the anamnesis information obtained from the families. Twins with different genders were automatically accepted as DZ twins. Zygosity was subsequently confirmed for 100 selected pairs of the same gender by analyzing 16 Short Tandem Repeat (STR) markers using the AmpFLSTR Identifiler Polymerase Chain Reaction (PCR) Amplification Kit (Applied Biosystems, Waltham, MA, USA) and subsequent fragment analysis. Out of 100 selected twin pairs, 34 were monozygotic and 66 were dizygotic. The final study sample included 149 pairs of MZ twins and 59 pairs of MZ twins who visited our clinic for their first dental examinations. The participants’ families signed written, voluntary, informed permission forms on behalf of both themselves and their kids.

### 2.3. Maternal Surveys and Clinical Information

During the oral examination of the twins participating in the study, data on the type of delivery, birth weights, gestational age, ETFPT, and duration of breastfeeding were collected from their parents using a detailed questionnaire.

#### 2.3.1. Birth Weight and Gestational Age

The children’s birth weights and gestational ages were categorized using the relevant World Health Organization standards [21,22,23].

For gestational age at birth, there were two categories: low gestational age (less than 37 weeks) and normal gestational age (at least 37 weeks). Moreover, perinatal information was gathered, including the gender and weight of the infants. The three categories used to classify birth weight were low birth weight (2500 g), normal birth weight (2500–4000 g), and macrosomia (>4000 g).

#### 2.3.2. Eruption Timing of the First Primary Tooth

ETFPT was evaluated within three groups: early eruption time (<6 months), normal eruption time (6–8 months), and late eruption time (>8 months) [21,23]. The collected genetic (MZ, DZ), maternal (type of delivery, gestational age), perinatal (infant’s birth weight and gender), and postnatal (duration of breastfeeding) information was analyzed, and the effects on children’s ETFPT were examined.

### 2.4. Statistical Analysis

Data were obtained from 404 individuals in 2 groups: the MZ and DZ twin groups. Multivariate statistical analyses were performed in the SmartPLS 3 package program, and descriptive statistics and graphics were prepared in the IBM SPSS Statistics 26 (IBM Corp., Armonk, NY, USA) package program.

**Figure 1:**
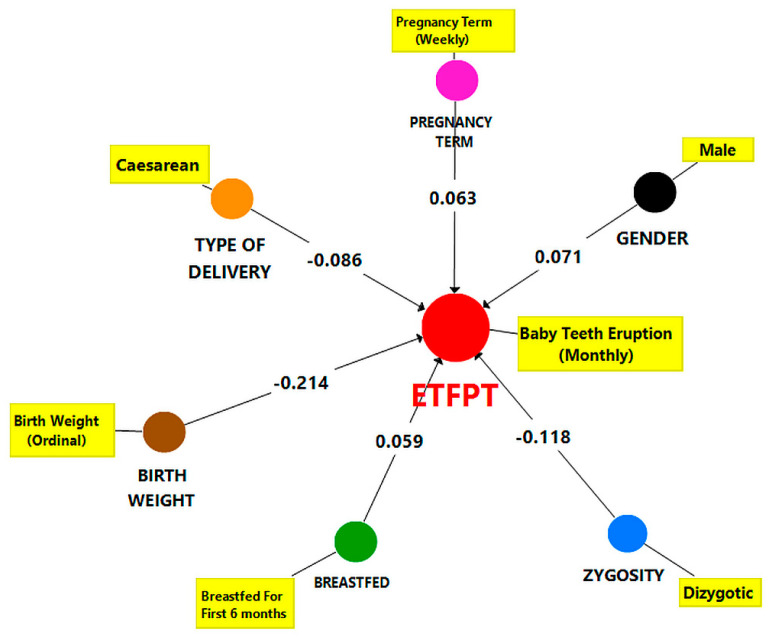
Partial Least Square Structural Equation Modeling (PLS-SEM) Source: Authors’ determined values by using the SmartPLS 3 software package.

The Partial Least Squares Structural Equation Model (PLS-SEM) technique was applied to the research model we designed (Figure 1). The hypotheses investigated in the model are as follows.

### 2.5. Hypotheses

The tested hypotheses are:

**Hypothesis** **1.** *There is a statistically significant relationship between zigosity and the ETFPT;*

**Hypothesis** **2.** *There is a statistically significant relationship between pregnancy and term (ETFPT);*

**Hypothesis** **3.** *There is a statistically significant relationship between gender and the ETFP;*

**Hypothesis** **4.** *There is a statistically significant relationship between breastfeeding and the ETFPT;*

**Hypothesis** **5.** *There is a statistically significant relationship between birth weight and the ETFPT;*

**Hypothesis** **6.** *There is a statistically significant relationship between the type of delivery and the ETFPT;*

**Hypothesis** **7.** *Zygosite has a moderating role in the relationship between pregnancy and term (ETFPT);*

**Hypothesis** **8.** *Zygosite has a moderating role in the relationship between breastfeeding and the ETFPT;*

**Hypothesis** **9.** *Zygosite has a moderating role in the relationship between birth weight and the ETFPT;*

**Hypothesis** **10.** *Zygosite has a moderating role in the relationship between the type of delivery and the ETFPT.*

Total effects were analyzed with the consistent PLS bootstrapping algorithm (Appendix A), and corrective effects were analyzed with the consistent multi-group analysis algorithm (MGAc). The PLS-SEM was used to analyze the relationships between maternal, perinatal, genetic, and postnatal variables and ETFPT in twin pairs. The estimated standard error values, t statistics, and confidence intervals in this technique are computed using nonparametric resampling (bootstrap analysis). It is suggested to resample 5000 times [24]. The model was run with 10,000 resamples and 5000 maximum iterations.

The heterotrait-monotrait ratio (HTMT) criterion is one of the methods recommended for assessing the discriminant validity of PLS-SEM models. Discriminant validity has been established if the HTMT values of the variables are less than 0.90 [25]. The HTMT ratio test findings of the present study varied from 0.00 to 0.54 (Table 1).

Therefore, the model provides discriminant validity. On the other hand, it was observed that all variance inflation factors (VIF) were below 10 (Table 2).

Accordingly, there were no problems with multicollinearity in the model [26]. Since there were no multicollinearity problems, the path coefficients were examined in the next step.

## 3. Results

A total of 404 twin siblings (202 pairs of twins) were enrolled in this study, including 204 boys and 200 girls. A total of 286 DZ twin pairs (70.8%) and 118 MZ twin pairs (29.2%) were examined genetically. The chronological ages of the siblings ranged from 3 to 15 years, with a mean age of 8.77 ± 3.22 years. ETFPT ranged from 0 months (natal teeth) to 24 months of age, with a mean of 6.92 ± 2.35 months (Table 3).

The data showed a statistically significant negative correlation between birth weight and ETFPT (B = −0.214, t = 2.964, *p* = 0.003) (Table 4).

The mean ETFPT was determined as 8.06 months, 6.96 months, and 6.59 months in infants born with very low, low, and normal birth weights, respectively (B = 0.286, *p* = 0.043) (Table 5).

**Figure 2:**
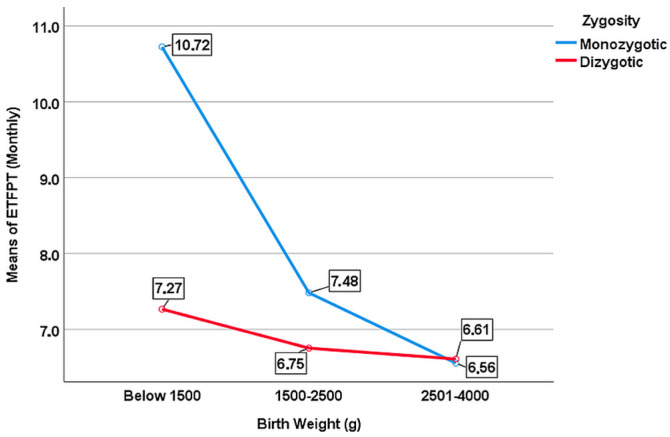
The moderating role of zygosity between birth weight and ETFPT.

MZ and DZ twins were coded as 0 and 1 for the zygosity variable, respectively, and so the obtained coefficient of 0.286 shows a positive effect for DZ twins. Therefore, the negative relationship between birth weight and ETFPT was stronger for MZ twins than it was for DZ twins (Figure 2).

The mean ETFPT was calculated as 10.72 months, 7.48 months, and 6.56 months in MZ twins born with very low, low, and normal birth weights, respectively. Among DZ twins, the mean ETFPT was 7.27 months, 6.75 months, and 6.61 months for children who were born with extremely low, low, and normal birth weights, respectively. The variation in the eruption time according to birth weight was much more pronounced among MZ twins. While variation in ETFPT depending on birth weight was significant in MZ twins (*p* = 0.001), it was not significant in DZ twins (*p* = 0.275) (Table 6). The f^2^ value of birth weight was calculated as 0.136 for MZ twins, 0.004 for DZ twins, and 0.032 for the entire study sample.

When the effect of breastfeeding, regardless of zygosity, on the ETFPT was examined, no statistically significant relationship was found (B = 0.059, t = 1.229, *p* = 0.219) (Table 4). However, when this relationship was examined separately for DZ and MZ twins, the statistical results differed. Thus, another moderating effect of zygosity was found between breastfeeding and ETFPT (B = −0.200, *p* = 0.029) (Table 5).

**Figure 3:**
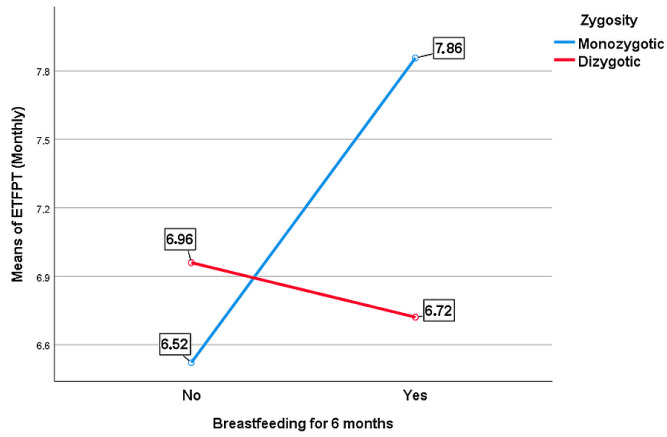
The moderating role of zygosity between breastfeeding and ETFPT.

The mean ETFPT was 6.52 months of age among MZ twins who were not breastfed for the first 6 months, and this value increased to 7.86 months for those who were breastfed for the first 6 months. Such an increase was not observed among DZ twins. While the mean ETFPT was 6.96 months of age among DZ twins who were not breastfed for the first 6 months, this value was 6.72 months for those who were breastfed for the first 6 months (Figure 3).

While the increase in ETFPT according to breastfeeding was significant in MZ twins (*p* = 0.019), the decrease in DZ twins was not significant (*p* = 0.422). The f^2^ value of breastfeeding was calculated as 0.027 for MZ twins and 0.003 for DZ twins. In addition, the total effect of zygosity on ETFPT in the model was statistically significant (B = −0.118, t = 2.23, *p* = 0.026) (Table 4).

While the mean of ETFPT for MZ twins was determined to be 7.31 months, this value was 6.75 months for DZ twins. The f^2^ value of zygosity was 0.015 for the entire study sample, including both MZ and DZ twins. No statistically significant relationship existed between gender or the length of the pregnancy and the ETFPT (*p* > 0.05). Likewise, no moderating effect of zygosity was found between type of delivery mode or pregnancy duration and ETFPT (*p* > 0.05).

## 4. Discussion

To investigate the influence of genetic variables on tooth eruption, the eruption periods of primary teeth in terms of zygosity were compared among pairs of twins in this study. At the same time, the potential moderating effects of zygosity on other factors that may affect tooth eruption were also evaluated, differently from other studies. It was found that ETFPT values were much closer to each other in MZ twin pairs. In addition, the effect of birth weight on tooth eruption times was stronger among MZ twins. This suggests that other factors exert stronger effects by reducing the impact of genetic factors on tooth eruption in MZ twins with higher genetic similarity ratios.

In this study, it was also seen that gender had no effect on ETFPT. In previous studies examining the effect of gender on differences in tooth eruption, conflicting findings were reported, with different studies reporting that tooth eruption was earlier in girls or boys [3,10]. There are also studies reporting that gender has no influence on the eruption timing of primary teeth [4,11]. Since the current study is a twin study, unlike previous research, the lack of effect of gender on ETFPT suggests that the effects of genetic factors may be greater [3,10].

The results of our study suggest that birth weight affects tooth development in the prenatal period and has an effect on the eruption timing of the primary tooth. Prenatal and postnatal nutrition are both important for the maturation of tooth enamel [27], and there is a relationship between low birth weight and dental hypoplasia [28,29]. It is thought that birth weight may indirectly affect tooth eruption age by affecting tooth development [27]. Our analysis results showed a negative relationship between birth weight and ETFPT, similar to previous studies [1,2,4,17,30]. Contrary to these studies, Alnemer et al. [3] found no relationship between the birth weight of the infant and the ETFPT.

Premature birth can negatively affect tooth development and cause delayed tooth eruption in infants [17,18,19]. According to Ntani et al. [31] and Wang et al. [32], delayed tooth eruption is strongly related to a shorter pregnancy. As a result of our analyses, contrary to those previous studies, an effect of the gestational period on ETFPT was not observed. This may be due to the fact that we chose pairs of twins as our study group. Our findings suggest that the effect of genetic similarity in twin patients on ETFPT is greater than the effect of the gestational period.

Studies on the type of delivery, which is one of the maternal factors, have mostly focused on enamel development [33,34,35]. It was stated that the type of delivery is effective on enamel development in a previous study [36]. Wu et al. [37] investigated the effects of delivery type on tooth eruption age in a study they conducted. In our study, no effects of the type of delivery were observed on ETFPT. Similarly, Mermarpour et al. [33] and Wu et al. [37] found no association between developmental enamel defects or tooth eruption age and delivery mode in their studies.

As a result of our analyses, no direct effect of breastfeeding for the first 6 months on ETFPT was observed for the entire study sample. However, when the children were evaluated according to zygosity status, zygosity was found to affect the relationship between postnatal nutrition and tooth eruption age. There was a relationship between breastfeeding duration and ETFPT among MZ twins, but such a relationship was not seen in the DZ group. This suggests that among DZ twins with more genetic differences, genetic factors have a greater effect on ETFPT as well as the duration of breastfeeding. When the path coefficients were examined, it was seen that the coefficient of the moderator effect of zygosity on the effect of birth weight (B = 0.286) was higher than the coefficient (B = −0.200) for the effect of the breastfeeding variable. Contrary to our study findings, Alnemer et al. [3] reported a positive relationship between breastfeeding and the eruption times of the first permanent teeth. Holman and Yamaguchi [38] also reported that delayed eruption of the upper incisors was observed in children who had not received breast milk. However, many studies have also reported that breastfeeding and its duration do not affect ETFPT [39,40]. In this twin study, the effect of 6 months of breastfeeding on the ETFPT values among twins was not significant in general; it was significant only for MZ twins, and that significance disappeared when DZ pairs of twins were included in the analysis. In this case, zygosity may moderate the relationship between breastfeeding and tooth eruption age, and that moderating effect may be variable.

The current investigation has a number of limitations. The use of supplements such as vitamin D and other vitamins was not evaluated within the category of nutrition. Another limitation is the fact that the effect of the amount of breast milk received by the infants according to percentiles could not be considered. Previous studies have also found earlier ETFPT in children born to mothers who smoked while they were pregnant, but this study did not evaluate the mothers’ habits, such as smoking during pregnancy. Furthermore, effects of other factors such as socioeconomic factors, maternal education level, and general maternal health were not examined here.

The contributions of this study to the literature can be listed as follows. First of all, this was a twin study in which genetic similarity was examined together with other factors of interest. This study was able to strengthen the causality among different factors by evaluating variables related to prenatal, postnatal, maternal, genetic characteristics, and ETFPT. Tooth eruption is clinically important for a child’s growth and development, the management of the occlusions, and treatment planning. Even if variations in eruption times are small, they may provide guidance for clinicians considering the factors that may affect the growth and development of children, and they may also shed light on conditions such as eruption problems and developmental diseases that may occur in the future. Therefore, it is important to investigate different determinants that may affect ETFPT.

## 5. Conclusions

In conclusion, the effects of breastfeeding and birth weight on ETFPT may differ according to zygosity in twins. MZ twins may tend to take longer to experience eruptions of their first primary teeth.

Bullet points:

- Birth weight affects tooth development in the prenatal period and has an effect on ETFPT;
- The variation of the eruption age according to birth weight was much more pronounced among MZ twins;
- A moderating effect of zygosity was found between breastfeeding and ETFPT.

## Figures and Tables

**Table 1:** Discriminant validity: heterotrait-monotrait ratio.

	Birth Weight	Breastfeeding	ETFPT	Gender	Pregnancy Term	Type of Delivery	Zygosity
Birth Weight							
Breastfeeding	0.083						
ETFPT	−0.162	0.045					
Gender	0.142	0.091	0.057				
Pregnancy Term	0.542	0.044	−0.028	0.094			
Type of Delivery	0.047	−0.037	−0.108	−0.050	−0.065		
Zygosity	−0.056	0.068	−0.109	−0.005	−0.081	0.022	

Abbreviations: ETFPT is the eruption timing of the first primary tooth (monthly).

**Table 2:** Collinearity assessment (inner VIF values).

	Criterion Variable
Predictors	ETFPT (Monthly)
Birth Weight	1.455
Breastfeeding	1.020
Gender	1.031
Pregnancy Term	1.437
Type of Delivery	1.019
Zygosity	1.013

Abbreviation: VIF is the variance inflation factor; ETFPT is the eruption timing of the first primary tooth (monthly).

**Table 3:** Descriptive statistics for siblings.

Number of Siblings: 404 Number of Twin Pairs: 202			
Variable	Group	n	%
Birth Weight (g)	Below 1500	39	9.7
	1500–2500	198	49.0
	2501–4000	167	41.3
Gender	Male	204	50.5
	Female	200	49.5
Zygosity	Monozygotic	118	29.2
	Dizygotic	286	70.8
Type of Birth	Normal	47	11.6
	Caesarean	357	88.4
Breastfeeding For First 6 Months	No	133	32.9
	Yes	251	62.1
	Missing	20	5.0
		**M**	**SD**
Chronological Age (Yearly)	Monozygotic	9.10	±3.04
	Dizygotic	8.63	±3.29
	Male	8.53	±3.27
	Female	9.01	±3.15
	Total	8.77	±3.22
ETFPT (Monthly)	Monozygotic	7.31	±3.25
	Dizygotic	6.75	±1.84
	Male	7.05	±2.74
	Female	6.78	±1.88
	Total	6.91	±2.35
Pregnancy Term (Weekly)	Monozygotic	36.02	±3.18
	Dizygotic	35.44	±3.26
	Male	35.91	±3.10
	Female	35.30	±3.37
	Total	35.61	±3.25

Abbreviations: ETFPT is the eruption timing of the first primary tooth. N is the sample size. M is the mean. SD is the standard deviation.

**Table 4:** Summary of relationships tested in the initial PLS path model.

Relationships	Original Sample (O)		SD	t	*p*	Confidence Intervals Bias Corrected		
						Bias	2.5%	97.5%
Birth Weight → ETFPT	−0.214	−0.214	−0.214	−0.214	−0.214	−0.214	−0.214	−0.214
Zygosity → ETFPT	−0.210	−0.210	−0.210	−0.210	−0.210	−0.210	−0.210	−0.210
Gender → ETFPT	0.072	0.072	0.072	0.072	0.072	0.072	0.072	0.072
Type of Delivery → ETFPT	2.964	2.964	2.964	2.964	2.964	2.964	2.964	2.964
Breastfeeding For First 6 Months → ETFPT	0.003 *	0.003 *	0.003 *	0.003 *	0.003 *	0.003 *	0.003 *	0.003 *
Pregnancy Term → ETFPT	0.004	0.004	0.004	0.004	0.004	0.004	0.004	0.004

Abbreviations: ETFPT is the eruption timing of the first primary tooth (monthly); SD is the standard deviation; * significant *p* value at the 0.05 level.

**Table 5:** Consistent multi-group analysis for zygosity.

	Dizygotic vs. Monozygotic	
	Path Coefficient	*p*
Breastfeeding For First 6 Months →ETFPT	−0.200	0.029 *
Birth Weight → ETFPT	0.286	0.043 *
Type of Delivery → ETFPT	0.118	0.365
Pregnancy Term (Weekly) → ETFPT	−0.022	0.845

Abbreviations: ETFPT is the eruption timing of the first primary tooth (monthly); * significant *p* value at the 0.05 level.

**Table 6:** Summary of testing the PLS path model in each subgroup for relationships moderated by zygosity.

Relationships	Zygosity Group	Original Sample (O)	Sample Mean (M)	SD	t	*p*	Confidence Intervals Bias Corrected		
							Bias	2.5%	97.5%
Breastfeeding → ETFPT	Dizygotic	−0.051	−0.050	0.064	0.789	0.422	−0.001	−0.176	0.073
	Monozygotic	0.149	0.148	0.063	2.366	0.019 *	−0.002	0.019	0.269
Birth Weight → ETFPT	Dizygotic	−0.082	−0.084	0.075	1.091	0.275	−0.002	−0.231	0.067
	Monozygotic	−0.368	−0.359	0.107	3.443	0.001 *	0.009	−0.551	−0.128

Abbreviations: ETFPT is the eruption timing of the first primary tooth (monthly); SD is the standard deviation; * significant *p* value at the 0.05 level.

## Data Availability

The data that support the conclusions of this investigation are accessible upon reasonable request from the corresponding author.

## Appendix A

The use of bootstrapping for nonparametric statistical inference has also been advocated as a descriptive tool and an internal replication mechanism for assessing the stability and reproducibility of sample results from an individual study. If the mean estimate is like our sample estimate and the standard deviation of estimates from the resampling is small, then we have some indication that the result is stable over many different configurations of subjects [41]. Based on that literature information, it can be said that the reported statistical results have replicability and stability.
